# Cerebrospinal fluid amyloid-β 42/40 ratio in clinical setting of memory centers: a multicentric study

**DOI:** 10.1186/s13195-015-0114-5

**Published:** 2015-06-01

**Authors:** Julien Dumurgier, Susanna Schraen, Audrey Gabelle, Olivier Vercruysse, Stéphanie Bombois, Jean-Louis Laplanche, Katell Peoc’h, Bernard Sablonnière, Ksenia V Kastanenka, Constance Delaby, Florence Pasquier, Jacques Touchon, Jacques Hugon, Claire Paquet, Sylvain Lehmann

**Affiliations:** Centre Mémoire Ressources Recherche (CM2R), Paris Nord Ile-de-France, Saint Louis, Lariboisière, Fernand-Widal Hospital, AP-HP, 200 rue du Faubourg Saint-Denis, 75010 Paris, France; INSERM U942, Biomarkers in CardioNeuroVascular diseases (Bio-CANVAS), University of Paris 7-Denis Diderot, Paris, France; Inserm, UMR 1172, JPArc, Faculté de Médecine, Université de Lille, 59045 Lille, France; Centre de Biologie-Pathologie, Centre Hospitalier Régional Universitaire, 59037 Lille, France; Centre Mémoire Ressources Recherche de Montpellier, Université de Montpellier, Montpellier, France; Biochimie-Protéomique Clinique - IRB - CCBHM, INSERM U1040, Université de Montpellier, Montpellier, France; Centre Mémoire Ressources Recherche, EA 1046, Centre Hospitalier Régional Universitaire de Lille, Lille, France; Service de Biochimie et Biologie Moléculaire, Hôpitaux universitaires Saint-Louis, Lariboisière, Fernand-Widal, Assistance Publique-Hôpitaux de Paris (AP-HP), PRES Sorbonne Paris Cité, Paris, France; Department of Neurology, MassGeneral Institute for Neurodegenerative Disease, Massachusetts General Hospital and Harvard Medical School, Charlestown, MA 02129 USA

## Abstract

**Introduction:**

The cerebrospinal fluid (CSF) biomarkers amyloid-β (Aβ), tau and phosphorylated tau (p-tau181) are now used for the diagnosis of Alzheimer’s disease (AD). Aβ40 is the most abundant Aβ peptide isoform in the CSF, and the Aβ 42/40 ratio has been proposed to better reflect brain amyloid production. However, its additional value in the clinical setting remains uncertain.

**Methods:**

A total of 367 subjects with cognitive disorders who underwent a lumbar puncture were prospectively included at three French memory centers (Paris-North, Lille and Montpellier; the PLM Study). The frequency of positive, negative and indeterminate CSF profiles were assessed by various methods, and their adequacies with the diagnosis of clinicians were tested using net reclassification improvement (NRI) analyses.

**Results:**

On the basis of local optimum cutoffs for Aβ42 and p-tau181, 22% of the explored patients had indeterminate CSF profiles. The systematic use of Aβ 42/40 ratio instead of Aβ42 levels alone decreased the number of indeterminate profiles (17%; *P* = 0.03), but it failed to improve the classification of subjects (NRI = −2.1%; *P* = 0.64). In contrast, the use of Aβ 42/40 ratio instead of Aβ42 levels alone in patients with a discrepancy between p-tau181 and Aβ42 led to a reduction by half of the number of indeterminate profiles (10%; *P* < 0.001) and was further in agreement with clinician diagnosis (NRI = 10.5%; *P* = 0.003).

**Conclusions:**

In patients with a discrepancy between CSF p-tau181 and CSF Aβ42, the assessment of Aβ 42/40 ratio led to a reliable biological conclusion in over 50% of cases that agreed with a clinician’s diagnosis.

**Electronic supplementary material:**

The online version of this article (doi:10.1186/s13195-015-0114-5) contains supplementary material, which is available to authorized users.

## Introduction

Dementia is a growing public health concern in aging populations. The number of people affected worldwide is set to reach 66 million by 2030 [1]. Alzheimer’s disease (AD) is the most frequent cause of dementia in the elderly. Clinical diagnosis is based on the presence of clinical symptoms, marked by a gradual onset of dementia in the presence of cognitive decline, when other putative disorders have been excluded [2]. Unfortunately, the accuracy of AD diagnosis based solely on clinical observation is associated with poor specificity. In most studies comparing clinical diagnosis with neuropathological findings, researchers have reported specificity lower than 70% [3]. Development of specific disease-modifying drugs and early diagnosis require an improvement in the accuracy of AD diagnosis.

The use of biomarkers has strengthened the link between clinical dementia and AD pathophysiology [2]. Currently, cerebrospinal fluid (CSF) biomarkers are commonly used in specialized memory clinics [4]. A characteristic feature of AD progression is a reduction in amyloid-β (Aβ) protein (that is, low CSF Aβ42 level) and an increase of neuronal degeneration biomarkers (that is, increase of CSF total tau and phosphorylated tau (p-tau181) levels) in CSF of subjects with AD [5]. The decrease in CSF Aβ42 levels appears to be an early phenomenon in AD progression and is evident over two decades prior to the first clinical sign [6]. Unfortunately, because CSF Aβ42 levels can also be low in non-AD patients, this biomarker alone is of limited use in a clinical setting [7,8]. The limited use of CSF biomarkers can lead to indeterminate results, revealing abnormal tau protein values and normal Aβ peptide levels or the inverse [2]. Furthermore, CSF biomarker results are also characterized by a large intersite variability [7] that requires the use of internally validated cutoff levels for each laboratory [9].

Aβ40 is the most abundant Aβ peptide isoform in the CSF. Compared with CSF Aβ42 levels alone, Aβ 42/40 ratio is suggested to account for the constitutive interindividual differences in total CSF Aβ peptide load between high- and low-amyloid-producing individuals [10]. The Aβ 42/40 ratio could also play an important role in Alzheimer’s disease pathogenesis [11]. However, the added value of the CSF Aβ40 level assessment remains controversial [12-15]. Recently, in a monocentric study, researchers reported that CSF Aβ 42/40 ratio may be of particular use in patients with ambiguous CSF results [16]. Therefore, the goal of our present study was to assess CSF Aβ 42/40 ratios and CSF Aβ42 levels alone to discriminate AD from non-AD patients in a large, prospective, multicentric population of patients. We hypothesized that the use of Aβ 42/40 ratio would reduce the frequency of inconclusive CSF results and would lead to a valid AD diagnosis.

## Methods

### Subjects

According to national recommendations, CSF analyses are routinely used during testing of patients with cognitive disorders in French memory centers [4,17]. Patients were recruited between 1 September 2012 and 1 September 2013 from three French clinical and research memory centers specializing in the care of patients with cognitive disorders. These three centers are based in Paris, Lille and Montpellier (the PLM Study) [18-20]. All patients who were prospectively included in the study had cognitive disorders and received a lumbar puncture. Patients with unknown clinical diagnoses were excluded. Patients with mild cognitive impairment (MCI) represented a heterogeneous group, including patients with and without AD-related brain lesions, and thus were excluded from this study.

All patients had a thorough examination, including clinical and neuropsychological evaluations, biological measurements and brain imaging. Patients with AD were included according to the criteria for probable AD as defined by the National Institute on Aging-Alzheimer’s Association [2]. On the basis of all available elements, which included the results for CSF biomarkers, patients were classified into two groups: AD and non-AD. Complex or unclear cases were discussed, and diagnoses were made by a multidisciplinary team of neurologists, geriatricians and neuropsychologists. Non-AD subjects included subjects with cognitive disorders other than AD (see Additional file 1), such as frontotemporal dementia, Lewy body disease, Parkinson’s disease, Creutzfeldt-Jakob disease and non-degenerative dementia (that is vascular dementia, alcohol-related dementia, normal pressure hydrocephalus, infectious disease and psychiatric disorders) [7].

This research study was approved by the local ethics committees of each hospital (see Additional file 2). All patients agreed to CSF collection, assessments and analyses by providing a written informed consent.

### Cerebrospinal fluid analysis

Lumbar punctures were performed on fasting patients within 1 month following their clinical diagnosis, usually between 9:00 am and 12:00 pm. In an effort to reduce the intersite variability in CSF readings previously reported [7], all three centers used a common 10-ml polypropylene tube to collect the CSF (catalog number 62.610.201; Sarstedt, Nümbrecht, Germany) [21]. Each CSF sample was transferred at 4°C to the corresponding local laboratory within 4 hours after collection and was then centrifuged at 1,000 *g* for 10 minutes at 4°C. A small amount of CSF was used to perform routine analyses, including total cell count, bacteriological examination and total protein and glucose levels. The CSF was aliquoted in 0.5-ml polypropylene tubes and stored at −80°C to await further analysis. CSF Aβ40, Aβ42, total tau and p-tau181 were measured in each local laboratory using a commercially available sandwich enzyme-linked immunosorbent assay (INNOTEST; Fujirebio Europe NV, Gent, Belgium) according to the manufacturer’s instructions. Concentrations of total tau greater than 1,200 pg/ml were well above the detection limit and thus were not recalculated after dilution because of the constraints in the procedure. The biological teams involved in CSF analyses were blinded to the clinical diagnoses.

### Cerebrospinal fluid cutoff determination and interpretation

CSF cutoffs used in the analyses were determined on the basis of the population included in the present study. We computed both overall and local cutoff receiver operating characteristic (ROC) curves using STATA software (v10, StataCorp, College Station, TX, USA). ROC curves were built for each biomarker by plotting sensitivity and 1 − specificity to discriminate AD from non-AD patients. Optimum cutoff values were determined using two methods: the highest Youden index (that is, sensitivity + specificity − 1) and lowest distance between ROC plot and point (0.1). We checked that both approaches led to similar findings, and the presented results correspond to the highest Youden index. STATA code used for the determination of optimum cutoff is detailed in the Additional file 3.

CSF results were defined as negative (that is, CSF p-tau181 below cutoff and CSF Aβ42 above cutoff) or positive (that is, CSF p-tau181 above cutoff and CSF Aβ42 below cutoff). An indeterminate CSF profile was defined by the association of one positive biomarker and one negative biomarker.

We chose to evaluate tau pathology based on CSF p-tau181 levels alone because p-tau181 and total tau levels were highly correlated in our sample (Pearson’s correlation coefficient = 0.87). Furthermore, p-tau181 has previously been reported to be more discriminant than total tau [22]. Analyses based on total tau led to findings and conclusions similar to those obtained with p-tau181.

### Statistical analysis

The subjects’ characteristics are presented overall, by diagnosis (AD vs. non-AD) and by CSF collection centers. The various groups (diagnoses, centers) were compared using analysis of variance for continuous variables and χ^2^ test for categorical variables.

We computed ROC curves to evaluate the capacity of each CSF biomarker to discriminate AD from non-AD subjects. Analyses were performed in the overall study population and then stratified by centers. Optimum cutoffs for each biomarker were defined using the highest Youden index to discriminate in the best possible manner AD from non-AD subjects, thus maximizing sensitivity and specificity of the analyses based on ROC curve analyses. Then we used local optimum cutoffs for further analysis because intersite differences were present.

To evaluate the interest of ratio Aβ 42/40 versus CSF Aβ42 alone, we compared three methods to interpret CSF results:

1. *Method 1*: interpretation based on CSF p-tau181 and on CSF Aβ42
2. *Method 2*: interpretation based on CSF p-tau181 and on Aβ 42/40 ratio
3. *Method 3*: a decisional algorithm based, in a first step, on CSF p-tau181 and on CSF Aβ42. Then, in case of discrepancy between p-tau181 and Aβ42, we used, in a second step, the Aβ 42/40 ratio in place of Aβ_1–42_

For all three methods, we first determined the percentage of AD, non-AD and indeterminate biological CSF profiles. Proportions of indeterminate profiles according to the methods were compared using the McNemar test. Then we used a net reclassification improvement (NRI) method to compare method 2 to method 1, and method 3 to method 1, among AD patients and non-AD patients (based on clinical diagnosis). Briefly, NRI is a statistical tool used to assess improvement in model performance offered by a new method of classification compared with a reference method [23,24]. The NRI compares the frequency of appropriate reclassification to inappropriate reclassification with the use of a new model of classification. The NRI is based on reclassification tables constructed separately for participants with or without the interest event (that is, diagnosis of AD or non-AD) and quantifies the correct movement in categories, up for events and down for non-events. Upward movement (up) is a change into a higher category, and downward movement (down) is a change into a lower category based on the new algorithm. The NRI is calculated as follows:

NRI = *P* (up | event) ‐ *P* (down | event) + *P* (down | non ‐ event) ‐ *P* (up | non ‐ event) [23]

The null hypothesis for NRI = 0 was tested using Z-statistics following McNemar asymptotic test for correlated proportions [22]. We provide an Excel file (Microsoft, Redmond, WA, USA) that allows the assessment of NRI with its standard error and its *P*-value [24].

All resulting *P*-values were two-tailed, and *P* ≤ 0.05 was considered statistically significant. Statistical analyses were performed using STATA version 10 and SAS version 9.2 (SAS Institute, Cary, NC, USA).

## Results

In this study, we enrolled a total of 367 participants who received a lumbar puncture between 1 September 2012 and 1 September 2013. The study was conducted at three study centers (Paris, n = 82 subjects; Lille, n = 124 subjects; Montpellier, n = 161 subjects). The subjects’ characteristics are summarized in Table 1. Compared with non-AD subjects, participants with AD were older, were more likely to be female and had lower Mini Mental State Examination scores. Furthermore, subjects with AD had higher CSF tau and p-tau181 levels, lower CSF Aβ42 levels and lower CSF Aβ 42/40 ratios. CSF Aβ40 levels tended to be higher in patients with AD compared with non-AD patients (*P* = 0.06), but this relationship was attenuated after adjustment for age and sex (*P* = 0.19).

**Table 1:** **Characteristics of the study population** ^**a**^

**Characteristics**	**Overall (N = 367)**	**AD (n = 160)**	**Non-AD (n = 207)**	***P*** **-value** ^**b**^
Age (yr), mean (SD)	65.9 (10.7)	67.9 (9.7)	64.5 (11.2)	0.002
Women, n (%)	190 (51.9)	97 (60.6)	93 (45.2)	0.003
MMSE, mean (SD)	22.2 (61.1)	20.3 (6.1)	23.6 (5.6)	<0.001
CSF biomarkers (pg/ml), mean (SD)				
Total tau	445.4 (334.8)	660.5 (349.5)	279.2 (202.9)	<0.001
pTau-181	62.8 (37.2)	89.7 (38.7)	42.0 (17.6)	<0.001
Aβ42	863.6 (357.3)	668.5 (297.3)	1,014.5 (325.9)	<0.001
Aβ40	15,426.6 (8,630.6)	16,383.6 (8,761.3)	14,686.9 (8,475.4)	0.06
Aβ 42/40	0.068 (0.047)	0.047 (0.022)	0.084 (0.054)	<0.001

^a^Aβ, Amyloid-beta; AD, Alzheimer’s disease; CSF, Cerebrospinal fluid; MMSE, Mini Mental State Examination; SD, Standard deviation. ^b^By χ^2^ test or analysis of variance.

The percentages of patients with AD across centers ranged from 31% to 59% (Table 2). There were no significant differences in age or sex between AD and non-AD patients across the three sites. In patients with AD, the Aβ 42/40 ratio was the only comparable biomarker between the three sites, with a mean value ranging from 4.4% to 4.9% (*P* = 0.39).

**Table 2:** **Intercenter characteristics** ^**a**^

**Characteristics**			**Center 1 (N = 82)**	**Center 2 (n = 124)**	**Center 3 (n = 161)**	***P*** **-value** ^**b**^
**AD patients**						
	Number of subjects (%)		37 (45.1)	73 (58.9)	50 (31.1)	<0.001
	Age (yr), mean (SD)		68.9 (8.6)	67.0 (9.5)	68.5 (10.7)	0.54
	Women, n (%)		27 (73.0)	39 (53.4)	31 (62.0)	0.14
	MMSE, mean (SD)		18.6 (6.5)	19.5 (6.3)	22.8 (4.9)	0.003
	CSF biomarkers, pg/ml, mean (SD)					
		Total tau	599.5 (281.2)	774.2 (370.7)	539.6 (315.2)	<0.001
		p-tau181	79.4 (24.4)	101.5 (42.2)	80.0 (39.2)	0.002
		Aβ42	656.9 (257.1)	603.2 (245.4)	772.5 (363.5)	0.008
		Aβ40	16,229.7 (6,252.8)	13,426.8 (5,250.9)	20,814.3 (12,114.6)	<0.001
		Aβ 42/40	0.044 (0.018)	0.049 (0.021)	0.045 (0.025)	0.39
**Non-AD patients**						
	Number of subjects (%)		45 (54.9)	51 (41.1)	111 (68.9)	<0.001
	Age (yr), mean (SD)		63.3 (10.7)	64.4 (10.6)	65.0 (11.8)	0.70
	Women, n (%)		17 (37.8)	20 (40.0)	56 (50.4)	0.25
	MMSE, mean (SD)		23.7 (4.9)	20.9 (6.1)	24.7 (5.3)	<0.001
	CSF biomarkers (pg/ml), mean (SD)					
		Total tau	201.5 (64.0)	283.8 (150.5)	308.6 (248.6)	0.01
		p-tau181	40.4 (12.8)	46.2 (15.8)	40.8 (19.7)	0.14
		Aβ42	984.3 (253.6)	987.0 (344.7)	1,039.3 (343.3)	0.50
		Aβ40	12,219.5 (4,775.2)	11,161.0 (4,955.5)	17307.2 (9,952.7)	<0.001
		Aβ 42/40	0.087 (0.024)	0.098 (0.041)	0.077 (0.066)	0.06

^a^Aβ, Amyloid-beta; AD, Alzheimer’s disease; CSF, Cerebrospinal fluid; MMSE, Mini Mental State Examination; p-tau181, tau phosphorylated at threonine 181; SD, Standard deviation. ^b^By χ^2^ test or analysis of variance.

The results of ROC curve analysis comparing AD with non-AD patients and optimum cutoffs are presented in Table 3. Area under the receiver operating characteristic curve (AUC) values for the various biomarkers can be used to discriminate between AD and non-AD patients. Across all three centers, tau (AUC range, 0.77 to 0.96) and p-tau181 levels (AUC range, 0.84 to 0.95) were more useful than Aβ42 levels (AUC range, 0.74 to 0.82) for AD diagnosis. Aβ 42/40 ratio outperformed Aβ42 in only one of the three centers, but not in the overall population (AUC = 0.81). Optimum cutoffs for CSF Aβ42 and for Aβ 42/40 ratio ranged from 737 pg/ml to 836 pg/ml and from 0.050 to 0.082, respectively.

**Table 3:** **Area under the receiver operating characteristic curve and optimal value to discriminate subjects with Alzheimer’s disease from those without** ^**a**^

**CSF biomarkers, pg/ml**		**AUC (SE)**	**Optimal value**	**Sensitivity**	**Specificity**	**Youden index**
Overall						
	Total tau	0.87 (0.02)	330	0.84	0.81	0.65
	p-tau181	0.90 (0.02)	62	0.79	0.91	0.70
	Aβ42	0.81 (0.02)	752	0.78	0.79	0.57
	Aβ 42/40	0.81 (0.02)	0.055	0.73	0.78	0.51
Center 1						
	Total tau	0.96 (0.01)	300	0.95	0.96	0.91
	pTau-181	0.95 (0.02)	58	0.86	0.91	0.77
	Aβ42	0.81 (0.05)	814	0.84	0.80	0.64
	Aβ 42/40	0.93 (0.03)	0.065	0.89	0.84	0.73
Center 2						
	Total tau	0.91 (0.03)	389	0.86	0.82	0.68
	p-tau181	0.91 (0.03)	64	0.85	0.86	0.71
	Aβ42	0.82 (0.04)	836	0.90	0.71	0.61
	Aβ 42/40	0.87 (0.03)	0.082	0.90	0.67	0.57
Center 3						
	Total tau	0.77 (0.04)	343	0.74	0.77	0.51
	p-tau181	0.84 (0.04)	62	0.72	0.92	0.64
	Aβ42	0.74 (0.05)	737	0.70	0.80	0.50
	Aβ 42/40	0.75 (0.04)	0.050	0.64	0.79	0.43

^a^Aβ, Amyloid-beta; AUC, Area under the receiver operating characteristic curve; CSF, Cerebrospinal fluid; p-tau181, tau phosphorylated at threonine 181; SE, Standard error.

**Figure 1:**
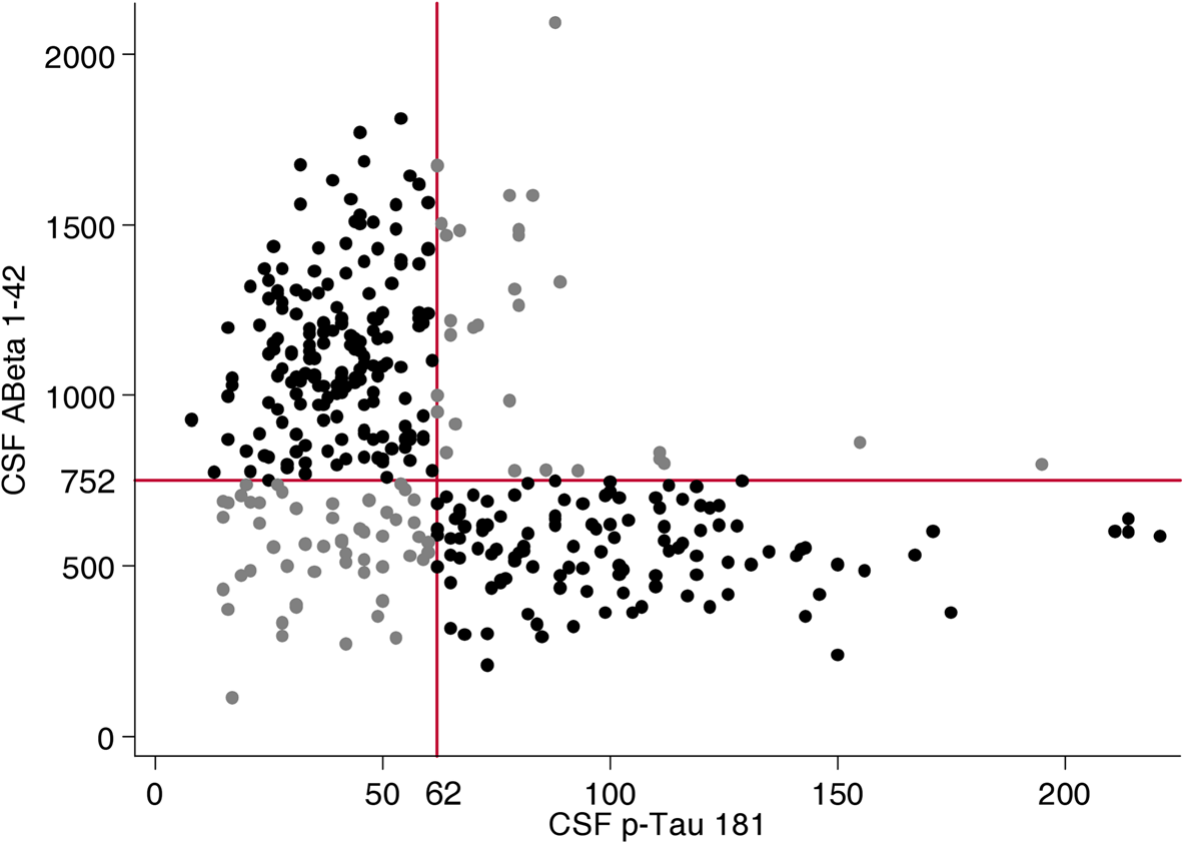
**Scatterplot of cerebrospinal fluid amyloid-β42 and phosphorylated tau values in the overall study population.** Black dots represent non-ambiguous cerebrospinal fluid (CSF) results (non-Alzheimer’s disease (non-AD) in the upper left quadrant, AD in the lower right quadrant). Gray dots represent indeterminate CSF results. Cutoffs for amyloid-β_42_ (Aβ42) and phosphorylated tau (p-tau181) were calculated in the overall population. Among the 367 patients, 83 (23%) had indeterminate CSF results.

Figure 1 illustrates the results of CSF biomarkers in the overall population according to CSF Aβ42 and CSF p-tau181 and per the global cutoffs based on the previous ROC curve analysis (overall population study). A total of 284 patients (77%) had determinate and definitive results using CSF Aβ_1–42_ and CSF p-tau181 levels (non-AD profile or AD-type profile), and 83 participants (23%) had indeterminate CSF results.

**Figure 2:**
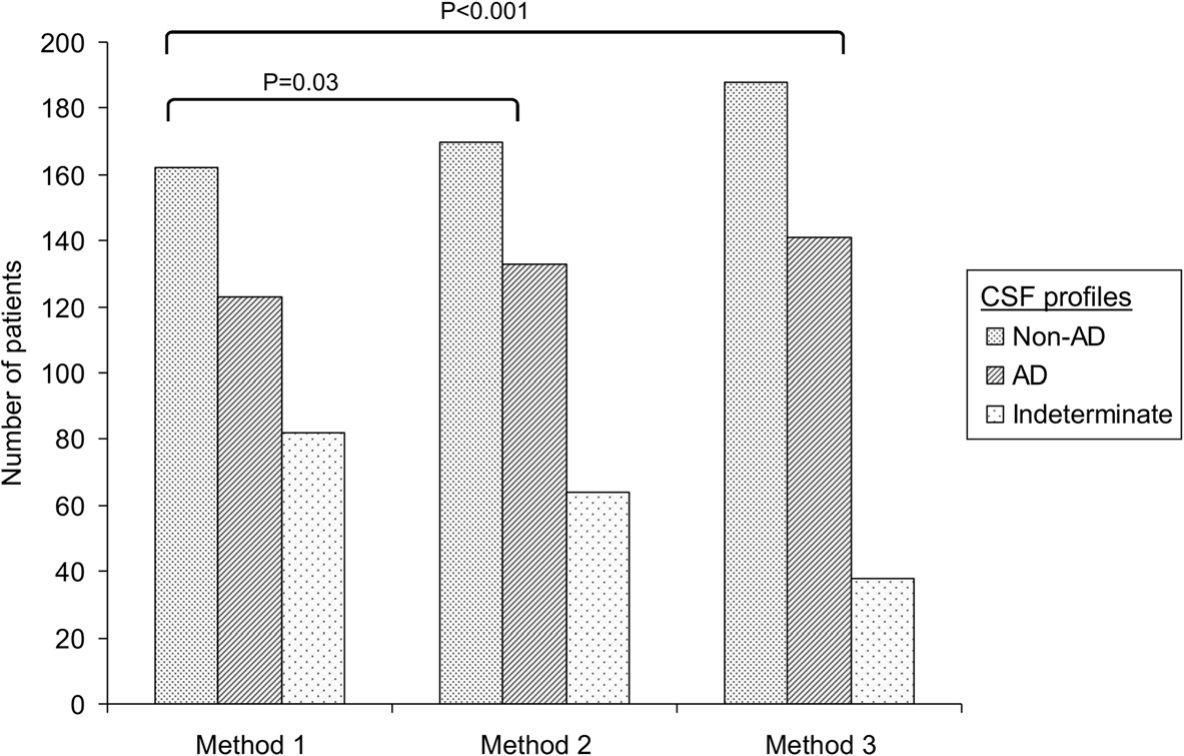
**Biological interpretation of cerebrospinal fluid results according to different methods.** Method 1: Cerebrospinal fluid (CSF) phosphorylated tau (p-tau181) and CSF amyloid-β_42_ (Aβ42). Method 2: CSF p-tau181 and Aβ 42/40 ratio. Method 3: First measure p-tau181 and CSF Aβ42, then use of Aβ 42/40 ratio instead of Aβ42 in case of discrepancy. Proportions of indeterminate profiles according to the methods were compared using the McNemar test.

Figure 2 illustrates the biological interpretation of CSF results according to three methods. (1) Interpretation based on CSF p-tau181 and CSF Aβ42 cutoffs (method 1) leads to 82 patients (22.3%) with indeterminate results. (2) Using CSF p-tau181 and Aβ 42/40 ratio (method 2) could reduce the number of indeterminate profiles to 64 patients (17.4%; *P* = 0.03 compared with method 1). (3) Use of CSF p-tau181 and CSF Aβ42, and then Aβ 42/40 ratio in case of discrepancy (method 3), attenuated the number of indeterminate results to 38 patients (10.4%; *P* < 0.001 compared with method 1).

**Figure 3:**
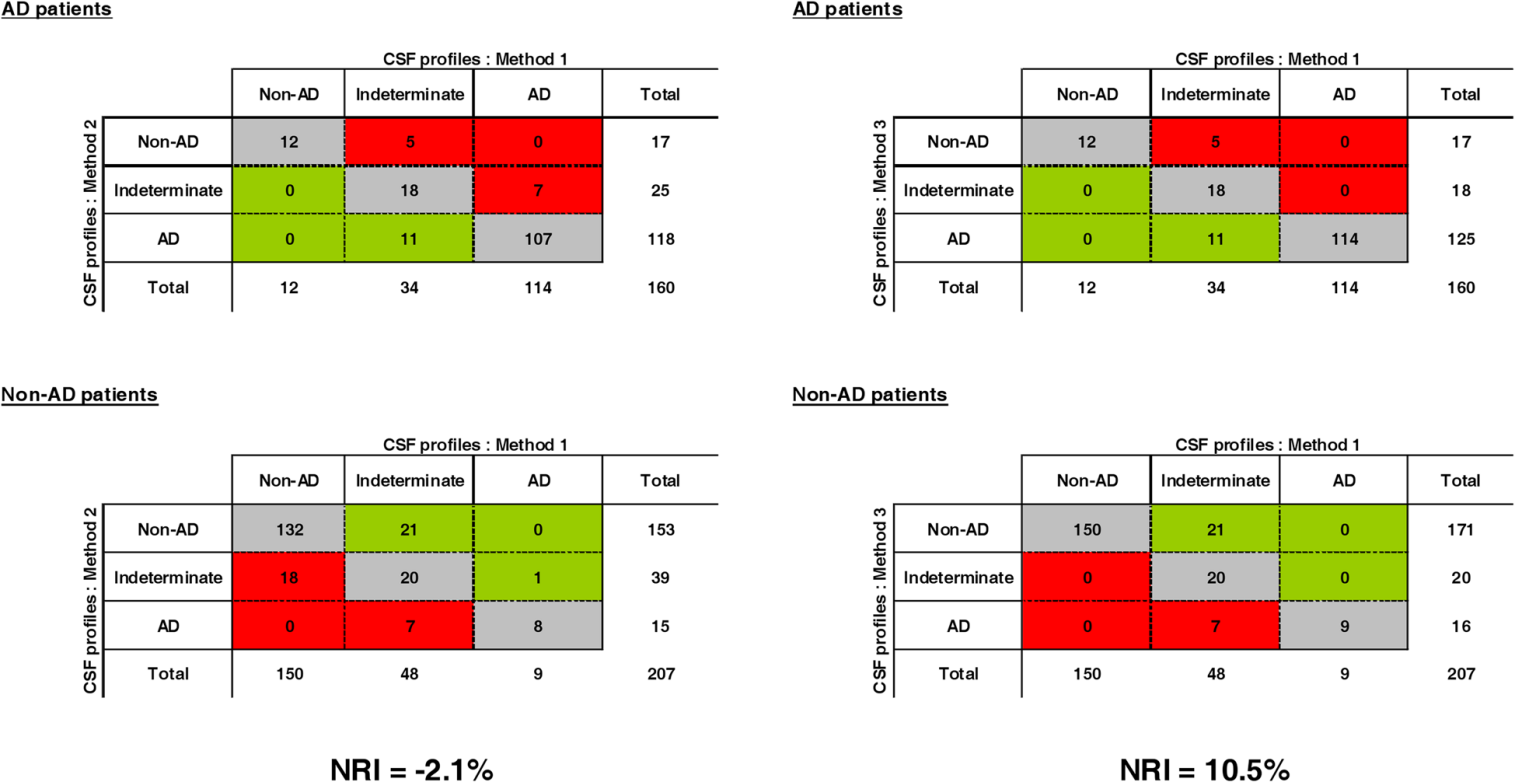
**Reclassification tables of different methods of cerebrospinal fluid interpretation among patients with Alzheimer’s disease and non-Alzheimer’s disease patients.** Method 1 (reference): cerebrospinal fluid (CSF) phosphorylated tau (p-tau181) and CSF amyloid-β_42_ (Aβ42). Method 2: CSF p-tau181 and Aβ 42/40 ratio. Method 3: First measure p-tau181 and CSF Aβ 42, then use Aβ 42/40 ratio instead of Aβ42 in case of discrepancy. Cells in green indicate an improvement compared with method 1, and cells in red indicate a worsening.

Figure 3 presents reclassification tables, comparing method 1 with methods 2 and 3 in patients with AD and non-AD patients. The systematic use of Aβ 42/40 ratio did not improve the classification of patients and tended even to be slightly worse than CSF Aβ42 alone with a NRI equal to −2.1% (standard error (SE) = 0.05, *P* = 0.64). By contrast, the use of Aβ 42/40 ratio only in patients with discordant results between p-tau181 and Aβ42 (method 3) improved the classification of patients, with a NRI at 10.5% (SE = 0.04, *P* = 0.003) compared with method 1.

## Discussion

In this clinically based, multicenter study, we evaluated the systematic assessment of CSF Aβ40 as part of a battery of CSF biomarkers measured in patients with cognitive disorders. We made the following observations:

- The Aβ 42/40 ratio was the only biomarker that was consistent between centers in AD and non-AD patients. We may therefore hypothesize that this ratio is less sensitive to preanalytical and analytical conditions.
- The systematic use of Aβ 42/40 ratio instead of Aβ42 decreased the number of indeterminate CSF profiles but failed to improve the classification of patients based on clinical diagnosis (NRI = −2.1%, *P* = 0.64).
- A strategy based on the use of Aβ 42/40 ratio instead of Aβ42 in patients with initially discrepant results between p-tau181 and Aβ42 led to a reduction of the number of indeterminate CSF profiles (10% vs. 22%) and improved the classification of patients (NRI = 10.5%, *P* = 0.003).

Consequently, the assessment of Aβ 42/40 ratio could be helpful and can be recommended as a second step when the initial CSF profile remains indeterminate.

Researchers in several previous studies have investigated the use of Aβ 42/40 ratio to differentiate patients with AD from patients with other cognitive disorders, and the reported results are heterogeneous. In a previous study, researchers found that Aβ 42/40 ratio outperformed Aβ42 alone in differentiating AD from non-AD patients, but the diagnostic performance of the combination of CSF tau and CSF Aβ42 was not improved by the use of Aβ 42/40 ratio instead of Aβ42 alone [15]. In another study, using a subset of autopsy-confirmed diagnoses, investigators found no overall difference in AUC between Aβ 42/40 ratio and Aβ42 alone to differentiate AD from non-AD patients. Those authors showed that the use of Aβ 42/40 ratio improved diagnostic accuracy in patients with intermediate levels of phosphorylated tau [12]. In other previous studies, researchers also found that the assessment of CSF Aβ40 may help to discriminate between AD patients and patients with frontotemporal lobar degeneration [25] or dementia with Lewy bodies [26] and Parkinson’s disease with dementia [27]. In contrast, the authors of another previous report found that the diagnostic accuracy of CSF Aβ42 was not different from the Aβ 42/40 ratio to discriminate AD patients from control subjects [14]. Controversial data have also been reported in patients with MCI, with some studies revealing that the Aβ 42/40 ratio may be useful to predict dementia conversion [28], whereas another did not [13].

Our findings confirm and strengthen the results of a recent study in which researchers reported that the use of the Aβ 42/40 ratio may contribute to decreases in the proportion of indeterminate CSF profiles in the clinical setting [16].

The proportion of patients with AD varied greatly at the three centers, reflecting the differences in recruitment and practices of memory clinics. Despite significant intercenter differences in reporting CSF Aβ40 and CSF Aβ42 levels, the mean Aβ 42/40 ratios were comparable across the three centers, ranging from 0.044 to 0.049 in patients with AD. We therefore hypothesize that the use of the CSF amyloid ratio could contribute to decreased preanalytical and analytical sources of variability among centers. Interestingly, we found that optimal cutoff for the ratio in the overall study population was equal to 0.055, which was comparable to the 0.057 cutoff recently reported by another team in a monocentric study [12].

Very few data are available concerning indeterminate profiles of CSF biomarkers. In our study population, on the basis of routine clinical practice, this situation was not rare, being observed in 22% of the patients even after optimization procedures using local optimum cutoffs. We have shown that Aβ 42/40 ratio instead of Aβ42 leads to a clear biological conclusion in more than 50% of these indeterminate cases. Reclassification analyses also showed that this approach is more congruent with the diagnosis by clinicians.

This study has several strengths, including its large size, its multicentric and prospective design, and the use of a common CSF polypropylene tubing at each of the three centers to standardize CSF evaluation. In addition, we used a NRI method that compares different strategies of biomarker analyses and is more precise than traditional analyses based on ROC curves. The main limitation of the results is the lack of neuropathological validations. Also, clinicians were not blinded to CSF results prior to clinical diagnosis, which may generate a circular reasoning bias in our findings. The absence of a centralized measurement for CSF biomarkers is another limitation, owing to the persistence of an intersite variability despite the use of a common tubing to collect CSF. Furthermore, the non-AD group was heterogeneous and included patients with cognitive disorders, of both psychiatric and neurologic origins. However, our study was aimed at reflecting the standard practice at memory clinics. The wide variety of patients with a number of diseases reflects the broad spectrum of cognitive complaints referred to memory centers. Finally, this study excluded patients with MCI. Inclusion of these patients in future studies will help to determine the links between the amyloid ratio and the rate of conversion from MCI to AD.

## Conclusions

Using a large prospective multicenter cohort of patients with cognitive disorders, we did not find an added value for the systematic assessment of the CSF Aβ 42/40 ratio. However, in cases of discrepancy between CSF p-tau and CSF Aβ42, the use of Aβ 42/40 ratio allowed reaching a biological conclusion in more than 50% of indeterminate results and improved the biological congruence with clinical diagnoses.

## Additional files

Additional file 1:
**List of local ethics committees that approved the research study.**
Additional file 2:
**STATA code used for determination of local optimum cutoffs.**
Additional file 3:
**Characteristics of non-AD patients.**

## Abbreviations

AD: Alzheimer’s disease
Aβ: Amyloid-β protein
Aβ40: 40–amino acid isoform of amyloid-β protein
Aβ42: 42–amino acid isoform of amyloid-β protein
AUC: Area under the receiver operating characteristic curve
CSF: Cerebrospinal fluid
MCI: Mild cognitive impairment
MMSE: Mini Mental State Examination
NRI: Net reclassification improvement
p-tau181: tau phosphorylated at threonine 181
ROC: Receiver operating characteristic
SD: Standard deviation
SE: Standard error
