# Supplementary material for: Cerebrospinal fluid amyloid-β 42/40 ratio in clinical setting of memory centers: a multicentric study
Source: Alzheimers Res Ther. 2015 Jun 1;7(1):30. doi: 10.1186/s13195-015-0114-5 (PMC4450486; doi:10.1186/s13195-015-0114-5)
Supplement: Additional file 1: — List of local ethics committees that approved the research study. [file 13195_2015_114_MOESM1_ESM.docx]

**Characteristics of non AD patients.**

| **Cognitive disorders** | **N** |
| --- | --- |
| Frontotemporal dementia | 46 |
| Psychiatric disorders | 42 |
| Parkinson disease | 24 |
| Lewy Body dementia | 21 |
| Vascular dementia | 13 |
| Alcohol dementia | 10 |
| HIV-associated neurocognitive disorder | 10 |
| Gait disorder | 9 |
| Multiple sclerosis | 9 |
| Progressive supranuclear palsy | 5 |
| Memory complaint | 4 |
| Creutzfeldt Jakob disease | 3 |
| Normal pressure hydrocephalus | 3 |
| Epilepsia | 3 |
| Post-chemotherapy cognitive impairment | 3 |
| Sjögren's syndrome | 2 |
