# Supplementary material for: Cerebrospinal fluid amyloid-β 42/40 ratio in clinical setting of memory centers: a multicentric study
Source: Alzheimers Res Ther. 2015 Jun 1;7(1):30. doi: 10.1186/s13195-015-0114-5 (PMC4450486; doi:10.1186/s13195-015-0114-5)
Supplement: Additional file 2: — STATA code used for determination of local optimum cutoffs. [file 13195_2015_114_MOESM2_ESM.docx]

**List of local ethics committees that approved the research study.**

- Paris : Ethics Committee of Paris University Hospital (Bichat Hospital), Paris, France.

- Lille : Comité de Protection des Personnes (CPP) Nord Ouest IV, Lille, France.

- Montpellier : Comité de Protection des Personnes Sud Méditérannée IV, Montpellier, France
