# Supplementary material for: Cerebrospinal fluid amyloid-β 42/40 ratio in clinical setting of memory centers: a multicentric study
Source: Alzheimers Res Ther. 2015 Jun 1;7(1):30. doi: 10.1186/s13195-015-0114-5 (PMC4450486; doi:10.1186/s13195-015-0114-5)
Supplement: Additional file 3: — Characteristics of non-AD patients. [file 13195_2015_114_MOESM3_ESM.docx]

**STATA code used for determination of local optimum cut-offs.**

Stata code :

use "data_base.dta", clear

senspec diag tau, sens(sens ) spec(spec)

gen youden= sens-(1-spec)

egen youdenmax= max(youden)

gen dist = sqrt((1-sens)^2 + (1-spec)^2)

egen distmin = min(dist)

list sens spec youdenmax dist tau if abs(youden -youdenmax)<0.0001

list sens spec youden distmin tau if abs(dist - distmin)<0.0001

**Index:**

**diag = variable corresponding to disease status : non AD = 0 ; AD = 1.**

**Sens = Sensitivity**

**Spec = Specificity**

**Youden = Youden index**

**Youdenmax = Maximum of Youden index**

**Dist = distance between ROC plot and point (0,1)**

**Distmin = minimum of distance between ROC plot and point (0,1).**
